# Sanghuang Tongxie Formula Ameliorates Insulin Resistance in *Drosophila* Through Regulating PI3K/Akt Signaling

**DOI:** 10.3389/fphar.2022.874180

**Published:** 2022-06-06

**Authors:** Xuqing Cao, Xiaojin La, Biwei Zhang, Zhigang Wang, Yinghong Li, Yanping Bo, Hong Chang, Xiujuan Gao, Chunyu Tian, Chenxi Wu, Ji-an Li

**Affiliations:** Hebei Key Laboratory of Integrated Traditional Chinese and Western Medicine for Diabetes and Its Complications, College of Traditional Chinese Medicine, North China University of Science and Technology, Tangshan, China

**Keywords:** Sanghuang Tongxie formula, insulin resistance, *Drosophila*, PI3K/AKT signaling, T2DM

## Abstract

Insulin resistance (IR) is a pivotal pathological characteristic that affects the occurrence and development of type 2 diabetes mellitus (T2DM). Thus, the effective control of IR is of great significance for diabetes prevention and treatment. Traditional Chinese medicine (TCM) represents a valuable tool handed down to the world by the Chinese nation and has a long history of use for diabetes clinical therapy. In this study, we focused on a self-drafted TCM-patented formula, Sanghuang Tongxie Formula (SHTXF), which exhibits clinical efficacy in the treatment of diabetes. To explore the effect and molecular mechanism of SHTXF on IR *in vivo*, *Drosophila melanogaster* was used and a (*Collagen*) *Cg* > InR^K1409A^ diabetic IR fly model was established. SHTXF water extract was found to contribute toward carbohydrate clearance from the circulating system by converting it into triglycerides (TAG), not glycogen, for nutrient storage. In addition, SHTXF activated phosphatidylinositol-3-kinase (PI3K) activity and improved protein kinase B (PKB, also termed Akt) phosphorylation. Finally, SHTXF promoted *Drosophila* Forkhead Box O (dFoxO) cytoplasmic localization and inhibited its transcriptional activity. Taken together, these findings not only highlight the positive role of SHTXF in ameliorating IR *via* the PI3K/Akt pathway but also provide potential drug targets and key insights for use in T2DM clinical treatment strategies.

## Introduction

Diabetes mellitus (DM) is a chronic metabolic disorder characterized by sustained hyperglycemia due to insulin resistance (IR) or deficiency. According to the latest data released by the IDF (10th edition), as many as 537 million adults (20–79 years old) worldwide have diabetes in 2021. Among these, type 2 diabetes mellitus (T2DM) accounts for 90%–95% of diabetic patients and is the result of the interaction between genetic and environmental factors (Pearson, 2019; Sun et al., 2022). With the rapid growth of the worldwide incidence, T2DM has become a serious threat to public health. IR is a significant pathological feature of T2DM, occurring throughout the occurrence and development of T2DM (Gutiérrez-Rodelo et al., 2017). The development of insulin resistance typically results in a compensatory increase in endogenous insulin production. The elevated levels of endogenous insulin, an anabolic hormone, are associated with insulin resistance and result in weight gain, which in turn exacerbates insulin resistance. This vicious cycle continues until pancreatic beta-cell activity can no longer adequately meet the insulin demand created by insulin resistance, resulting in hyperglycemia. With the continued mismatch between insulin demand and insulin production, glycemic levels rise to levels consistent with T2DM (Freeman and Pennings, 2020). Therefore, the mitigation, prevention, and control of metabolic abnormalities caused by IR are the main measures for the treatment of T2DM. Although great progress has been made in the study of oral hypoglycemic drugs and insulin, the effective rate of some general medicines for treating diabetes is only 41%, which is far from satisfactory (Nagpal and Bhartia, 2006; Agarwal et al., 2014). In addition, an increasing number of potential toxicities and side effects have been observed in drugs for the treatment of diabetes, including weight gain, bone loss, and increased risk of cardiovascular disease (Valerón and de Pablos-Velasco, 2013; Pang et al., 2015).

Traditional Chinese medicine (TCM) has a long history of use in the prevention and treatment of diabetes in China and other East Asian regions. In contrast to Western medicines, TCM exerts its action *via* multiple ingredients and multiple target regulatory networks, based on their composition, the functional characteristics of targets, and overall regulation (Tian et al., 2019). Moreover, TCM has the advantages of having low toxicity, fewer side effects, and low cost. Therefore, the research and development of TCM for the prevention and treatment of diabetes is an important area of interest. Sanghuang Tongxie Formula (SHTXF) is a self-drafted patent prescription by Professor Ji-an Li, created based on a combination of the theory of TCM and clinical experience in the treatment of diabetes (Chinese invention patent no. ZL201510724687.8). This formula contains 10 herbal drugs and has the function of clearing the stomach and purging the lung, regulating Qi and turbidity, tonifying the spleen, and benefiting the kidney. The details on the drug composition, as well as the amount of each herb in one dose, are provided in Table 1. In this study, SHTXF was evaluated for its molecular mechanism in the treatment of IR diabetes.

**TABLE 1:** Details of SHTXF (one dose).

Latin name (Chinese name)	Medicinal parts	Manufacturers	Batch number	Amount in application(g)	Herbarium number
*Morus alba L*. [Moraceae] (Sang Baipi)	Bark	Beijing Bencaofangyuan Pharmaceutical Co., Ltd.	20191120	15.0	NCSTTCM-2020017
*Coptis chinensis Franch.* [Ranunculaceae] (Huang Lian)	Root	Beijing Tongrentang Health Pharmaceutical Co., Ltd	26819202	15.0	NCSTTCM-2020018
*Magnolia officinalis Rehder and E. H. Wilson* [Magnoliaceae] (Hou Po)	Bark	Beijing Tongrentang Health Pharmaceutical Co., Ltd	190901	10.0	NCSTTCM-2020019
*Pueraria montana* var. *Thomsonii (Benth.) M.R.Almeida* [Fabaceae] (Ge Gen)	Root	Beijing Tongrentang Health Pharmaceutical Co., Ltd	190901	5.0	NCSTTCM-2020020
*Astragalus mongholicus Bunge* [Fabaceae] (Huang Qi)	Root	Beijing Bencaofangyuan Pharmaceutical Co., Ltd.	20180613	5.0	NCSTTCM-2020021
*Cornus officinalis Siebold & Zucc.* [Cornaceae] (Shan Zhuyu)	Fruit	Beijing Tongrentang(Fu zhou) Health Pharmaceutical Co., Ltd	20200801	5.0	NCSTTCM-2020022
*Raphanus raphanistrum subsp. Sativus (L.) Domin* [Brassicaceae] (Lai Fuzi)	Seed	Beijing Bencaofangyuan Pharmaceutical Co., Ltd.	20200202	5.0	NCSTTCM-2020023
*Anemarrhena asphodeloides Bunge* [Asparagaceae] (Zhi Mu)	Root	Beijing Bencaofangyuan Pharmaceutical Co., Ltd.	20200621	5.0	NCSTTCM-2020024
*Polygonatum odoratum (Mill.) Druce* [Asparagaceae] (Yu Zhu)	Root	Beijing Bencaofangyuan Pharmaceutical Co., Ltd.	20200605	5.0	NCSTTCM-2020025
*Atractylodes lancea (Thunb.) DC.* [Asteraceae] (Cang Zhu)	Root	Beijing Bencaofangyuan Pharmaceutical Co., Ltd.	20200818	5.0	NCSTTCM-2020026

Insulin resistance is a key risk factor and an important source of T2DM, the development of which involves the dysregulation of various signaling pathways, such as the PI3K/Akt, protein kinase C (PKC), AMP-activated protein kinase (AMPK), and nuclear factor κB (NF-κB) pathway (Yaribeygi et al., 2019). PI3K/Akt signaling is a highly conserved and important cellular transduction pathway involved in the regulation of glucose transport, glycogen synthesis, glycolysis and gluconeogenesis, protein synthesis, and lipolysis, playing a vital role in cell migration, differentiation, and apoptosis (Huang et al., 2018). For the past two decades, *Drosophila melanogaster* has been shown to be an appropriate model organism for T2DM modeling (Musselman et al., 2011; Pasco and Léopold, 2012) in view of the conserved insulin-like peptides (dILPs)/insulin receptor (InR)/insulin receptor substrate (IRS) module and downstream components involved in glycolipid homeostasis. Thanks to powerful genetic tools and reduced genome redundancy (Reiter et al., 2001; Garofalo, 2002), flies have been found to have a nutrition-sensing mode and intracellular signaling pathway (e.g., PI3K/Akt axis) similar to that of mammals at the cellular level. Phosphatidylinositol-3-kinase (PI3K) phosphorylates the D3 hydroxyl group of the inositol ring on PI to form the second messenger phosphatidylinositol-3,4,5-triphosphate (PIP3), which promotes protein kinase B (Akt/PKB) recruitment at the cell membrane and induces allosteric activation (Britton et al., 2002). Finally, activated Akt can lead to the phosphorylation of a large number of important substrates, including the transcription factor FoxO, to execute specific physiological activities (Klöting and Blüher, 2005).

In this study, we established a *Cg* > InR^K1409A^ diabetes fly model to analyze the effects of SHTXF extract on InR/PI3K/Akt-mediated IR in *Drosophila*, providing a potential molecular mechanism and theoretical basis for SHTXF in the clinical treatment of T2DM.

## Materials and Methods

### Sanghuang Tongxie Formula Extract Preparation

**FIGURE 1:**
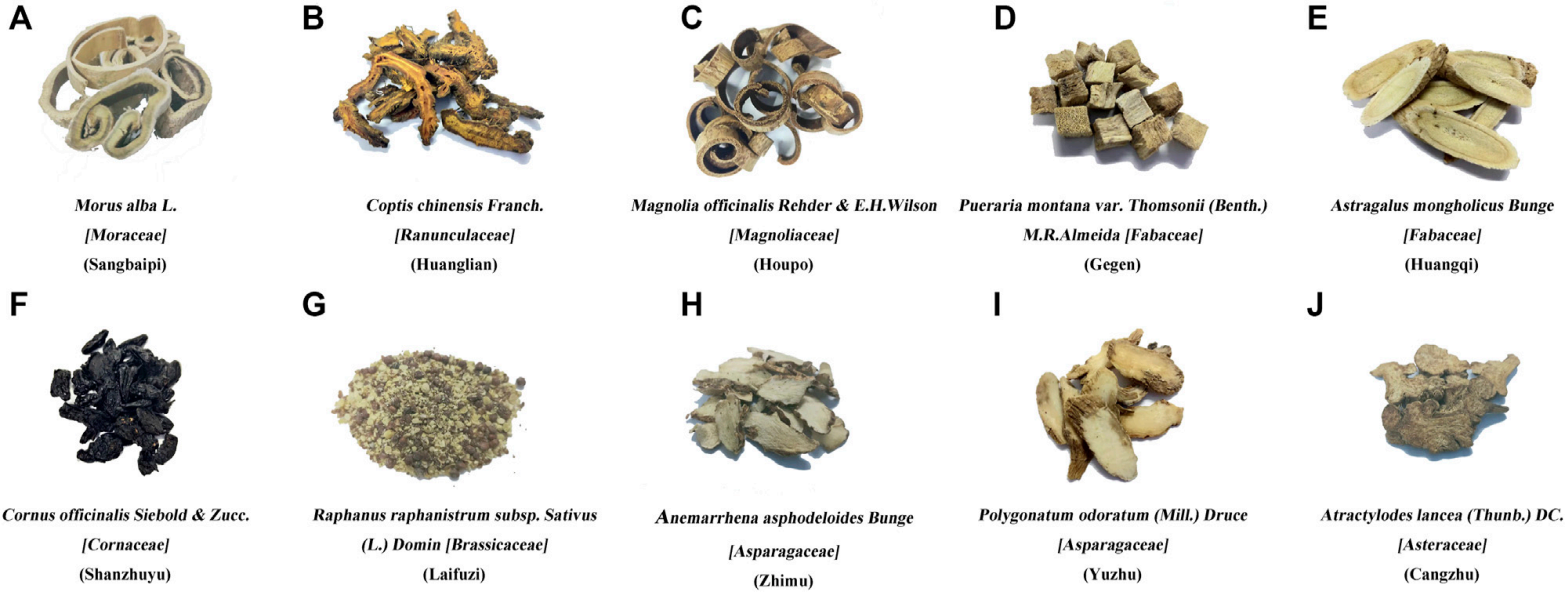
Composition of Sanghuang Tongxie Formula. The photographs show the composition of SHTXF (Sanghuang Tongxie formula). *Morus alba L*. [Moraceae] Sangbaipi, **(A)**, *Coptis chinensis Franch.* [Ranunculaceae] [Huanglian, **(B)**], *Magnolia officinalis Rehder and E. H. Wilson* [Magnoliaceae] [Houpo, **(C)**], *and Pueraria montana* var. *Thomsoni* (*Benth.*) *M.R.Almeida* [Fabaceae] [Gegen, **(D)]**, *Astragalus mongholicus Bunge* [Fabaceae] [Huangqi, **(E)**], *Cornus officinalis Siebold & Zucc.* [Cornaceae] [Shanzhuyu, **(F)**], *Raphanus raphanistrum subsp. Sativus* (*L.*) *Domin* [Brassicaceae] [Laifuzi, **(G)**], *Anemarrhena asphodeloides Bunge* [Asparagaceae]; Zhimu, **(H)**, *Polygonatum odoratum* (*Mill.*) *Druce* [Asparagaceae] [Yuzhu, **(I)**], and *Atractylodis rhizama* [Cangzhu, **(J)**].

To prepare Sanghuang Tongxie Formula (SHTXF), ten herbs (*Morus alba L*. [Moraceae] and *Coptis chinensis Franch*. [Ranunculaceae], *Magnolia officinalis Rehder & E. H. Wilson* [Magnoliaceae], *Pueraria montana* var. *Thomsoni* (*Benth.*) *M.R.Almeida* [Fabaceae], *Astragalus mongholicus Bunge* [Fabaceae], *Cornus officinalis Siebold & Zucc*. [Cornaceae], *Raphanus raphanistrum subsp*. *Sativus* (*L.*)*. Domina* [Brassicaceae], *Anemarrhena asphodeloides Bunge* [Asparagaceae], *Polygonatum odoratum* (*Mill.*) *Druce* [Asparagaceae], *and Atractylodes lancea* (*Thunb.*) *DC*. [Asteraceae]) (Figure 1) were purchased from Beijing Tongrentang Tangshan Chain Store Drug Store Co., Ltd., and authenticated by Prof. Chunyu Tian at the College of Traditional Chinese Medicine, North China University of Science and Technology. The manufacturer, batch number, and weight of each crude herb used for each dose are listed in Table 1.

Combined drugs of one dose (75 g) were weighed and soaked in 1.5 L of double-distilled water (ddH_2_O) for 12 h (Wang et al., 2020), followed by heating for 2 h. Next, the extract was separated and centrifuged at 4,200 × *g* for 30 min at 4°C, and the residue was collected to repeat the abovementioned steps. The two supernatants were then mixed and filtered by vacuum filtration with a 0.45-µm filter. Finally, the pumped liquid was placed in a beaker with medium fire to obtain a stock extract at a concentration of 0.5 g/ml, when the volume of the liquid reached 150 ml (Supplementary Figure S1). The prepared extract was directly mixed in the regular food to obtain a final concentration of 0.0125, 0.025, 0.05, or 0.1 g/ml SHTXF medium. Water alone was used as a control food.

### Quality Control by High-Performance Liquid Chromatography

Standard chemicals (berberine, magnolol, and morroniside) were purchased from the National Institutes for Food and Drug Control (NIFDC). Briefly, the quality of SHTXF was determined using a high-performance liquid chromatography (HPLC) system equipped with a UV-visible detector (Kamal et al., 2015). The analysis was performed using a Shimadzu LC-20A instrument. All chromatographic separations were carried out on an Agilent Eclipse XDB-C18 column (4.6 mm × 250 mm, 5 μm) at 35°C. The mobile phase for berberine was acetonitrile (A) and 0.1% phosphoric acid (B) (A: B = 50:50) by adding 0.1 g of sodium dodecyl sulfonate per 100 ml. A gradient system consisting of methanol (C) and water (D) (C:D = 78:22) was used to elute the magnolol. The mobile phase of morroniside was acetonitrile (E) and 0.3% phosphoric acid solution (F), and the elution gradients were 0–20 min (E: 7%, F: 93%) and 20–50 min (E: 7%–20%, F: 93%–80%).

### *Drosophila* Strains

Flies were maintained on a diet of cornmeal and sucrose medium (100°ml medium preparation: 5 g of sucrose, 1 g of agar, 6 g of cornflour, 3 g of yeast, 0.6 ml of propionic acid, and 100 ml of ultra-pure water) at 25°C in a 12 h light-dark cycle incubator with 50%–60% relative humidity. The *Drosophila* stocks used included: *w* ^*1118*^ (#3605), *Cg*-Gal4 (#7011), *UAS*-InR^K1409A^ (#8253), tGPH (#8164), and *dFoxO*-GFP (#37585) obtained from Bloomington *Drosophila* stock center (BDSC) in Indiana University. The crossing scheme for all the groups is shown in Supplementary Figure S2. For all fly crossing assays, healthy, unmated male and female parents were randomly assigned to different groups.

### Food Intake

Early third-instar larvae were starved for 2 h under adverse food conditions (0.8% agar in PBS) and then transferred to fresh dye-containing food (0.5% Brilliant Blue FCF) for 20 min. After feeding, the larvae were washed with phosphate-buffered saline (PBS), dried on tissue paper, and homogenized in an Eppendorf (EP) tube containing 100 µl of lysis buffer using a micro high-speed homogenizer (HR-68; Shanghai Hutong Industrial Co., Ltd.). After boiling and centrifuging for 3 min, followed by clearing by a high-speed refrigerated centrifuge (5430R; Eppendorf), 2 µl of the supernatant was analyzed with a spectrophotometer at 630 nm. A small amount of dye-containing food was weighed, processed as described above, and used as the standard to calculate the amount of ingested food (Matsuda et al., 2015).

### Hemolymph Glucose and Trehalose Measurement

The larvae were collected, rinsed with phosphate-buffered saline (PBS), and dried on tissue paper. The cuticle was carefully torn to release hemolymph on the parafilm membrane. Two microliters of hemolymph were collected using a micropipette, diluted 20 times with cold PBS, centrifuged for 3 min, and cleared by a high-speed refrigerated centrifuge. The supernatant was boiled at 75°C for 10 min and cleared by centrifugation. Next, glucose was measured after 15 min of incubation at 37°C using HK reagent (G3293; Sigma). A plate reader (M200PRD; Tecan) was used to measure absorbance at 340 nm.

Trehalose was converted using porcine trehalase (T8778; Sigma) overnight at 37°C, and the total amount of glucose was measured in the same way. The hemolymph trehalose concentration was determined by subtracting the value of free glucose from the untreated sample.

### Protein, Triglyceride, and Glycogen Measurement

Six third-instar larvae were collected and rinsed several times with PBS to remove traces of food. The frozen samples were homogenized using a pellet pestle in 200 µl of cold 0.05% PBST (PBS + 0.05% Tween-20) on ice, immediately heat-inactivated at 75°C for 10 min, and then cooled to room temperature (RT). This was followed by homogenization in an EP tube containing 100 µl of lysis buffer using a high-speed homogenizer. Next, 10 μl of homogenate were centrifuged for 3 min, cleared by a high-speed refrigerated centrifuge, and used for the measurement of free glycerol. Ten microliters of homogenate were used to determine the protein content using a bicinchoninic acid assay (BCA) protein assay kit (P0010; Beyotime).

Ten microliters of the homogenate were mixed with 10 µl of a triglyceride reagent (T2449; Sigma) including lipase for hydrolysis of triglycerides to glycerol, incubated at 37°C for 60 min, centrifuged for 3 min, and cleared by a high-speed refrigerated centrifuge. Ten microliters of the supernatant were used for the measurement of triglycerides (TAG) using free glycerol reagent (F6428; Sigma) at 540 nm. A triolein-equivalent glycerol standard (G7793; Sigma) was used as the standard. The TAG concentration for each sample was determined by subtracting the values of free glycerol from the untreated samples.

Five microliters of the sample were incubated with 10 µl of buffer A [5 mM Tris-HCl (pH 6.6), 137 mM NaCl, and 2.7 mM KCl] containing amyloglucosidase (10115-1 g; Sigma) at 50°C for 60 min to digest glycogen. Ten microliters of the sample were incubated with 5 µl of buffer A without enzymes in parallel for the determination of glucose levels. Glycogen was used as a standard (10901393001; Sigma). The glycogen concentration for each sample was determined by subtracting the values of free glucose in the untreated samples. The amounts of TAG and glycogen per fly were normalized to soluble protein levels per fly (Li and Tennessen, 2017).

### Lipid Droplet Staining

For Nile red staining, larvae were quickly inverted and dissected in 0.3% PBST. The tissues were incubated for 5 min with 0.001% Nile Red (72485; Sigma) in 0.3% PBST and washed twice with PBS at room temperature. The stained samples were mounted in 75% glycerol for microscopic analysis.

### Immunostaining

The larval discs were dissected and fixed in 4% formaldehyde for 20 min at room temperature (RT). After several washes with 0.3% (v/v) PBST, the discs were stained with primary antibodies at 4°C overnight and then with secondary antibodies at RT for 2 h. The following antibodies were used: mouse anti-Dlg1 (1:100) (4F3 anti-discs large; Developmental Studies Hybridoma Bank), rabbit anti-Akt (1:400) [4,691; cell signaling technology (CST)], rabbit anti-p-Akt (1:400) (4,060; CST), goat anti-mouse-cyanine3 (Cy3) (1:1000) (A10521; Life Technologies), and goat anti-rabbit Alexa 488 (1:1000) (4,412; CST). Vectashield medium (H-1500; Vector Laboratories) with DAPI (4’,6-diamidino-2-phenylindole) was used for mounting.

### Western Blotting

Whole larvae were lysed in radioimmunoprecipitation assay (RIPA) buffer (P0013; Beyotime) containing phosphatase inhibitor cocktail A (P1082; Beyotime) and phenylmethanesulfonyl fluoride (PMSF). Equal amounts of protein (10–30 µg, measured by BCA assay) were separated by sodium dodecyl sulfate-polyacrylamide gel electrophoresis (SDS-PAGE), transferred to a polyvinylidene fluoride (PVDF) membrane, and subjected to the standard western blot protocol, as previously described (Roth et al., 2018). The antibodies used in this study were rabbit anti-Akt (1:1000) (4,691; CST), rabbit anti-p-Akt (1:1000) (4,060; CST), rabbit anti-α-tubulin (1:1000) (2,125; CST), and goat anti-rabbit IgG (H + L, HRP) (1:10000) (5220-0336; Sera Care).

### qRT-PCR

TRIzol (Invitrogen) and PureLink^TM^ RNA Mini Kit (12183018A; Life Technologies) were used to isolate total RNA from third-instar larvae of the indicated groups, followed by qRT-PCR as previously described (Wang et al., 2003). The relative amounts of transcripts were calculated using the comparative *C*_*T*_ method with *ribosomal protein 49* (*rp49*) as the reference gene. The following primers were used:

For *rp49* Sense: 5’- TCC​TAC​CAG​TTC​AAG​ATG​AC—3’
Antisense: 5’- TCC​TAC​CAG​TTC​AAG​ATG​AC—3’
For *4EBP* Sense: 5’- TGA​TCA​CCA​GGA​AGG​TTG​TCA​TCT​C—3’
Antisense: 5’- GAG​CCA​CGG​AGA​TTC​TTC​ATG​AAA​G—3’

### Statistical Analysis

All data were verified using at least three independent experiments. The results were processed using GraphPad Prism 8.0 software as bar graphs. One-way analysis of variance (ANOVA) with Bonferroni’s multiple comparison test was employed for statistical analysis. The *p* value was set at *p* < 0.05, and the center value was considered the mean. The error bars indicate standard deviation. “ns” denotes “not significant” values (*p* ≥ 0.05); *∗p <* 0.05; *∗∗p <* 0.01; *∗∗∗p <* 0.001, and *∗∗∗∗p <* 0.0001.

## Results

### Sanghuang Tongxie Formula Preparation and High-Performance Liquid Chromatography Analysis

**FIGURE 2:**
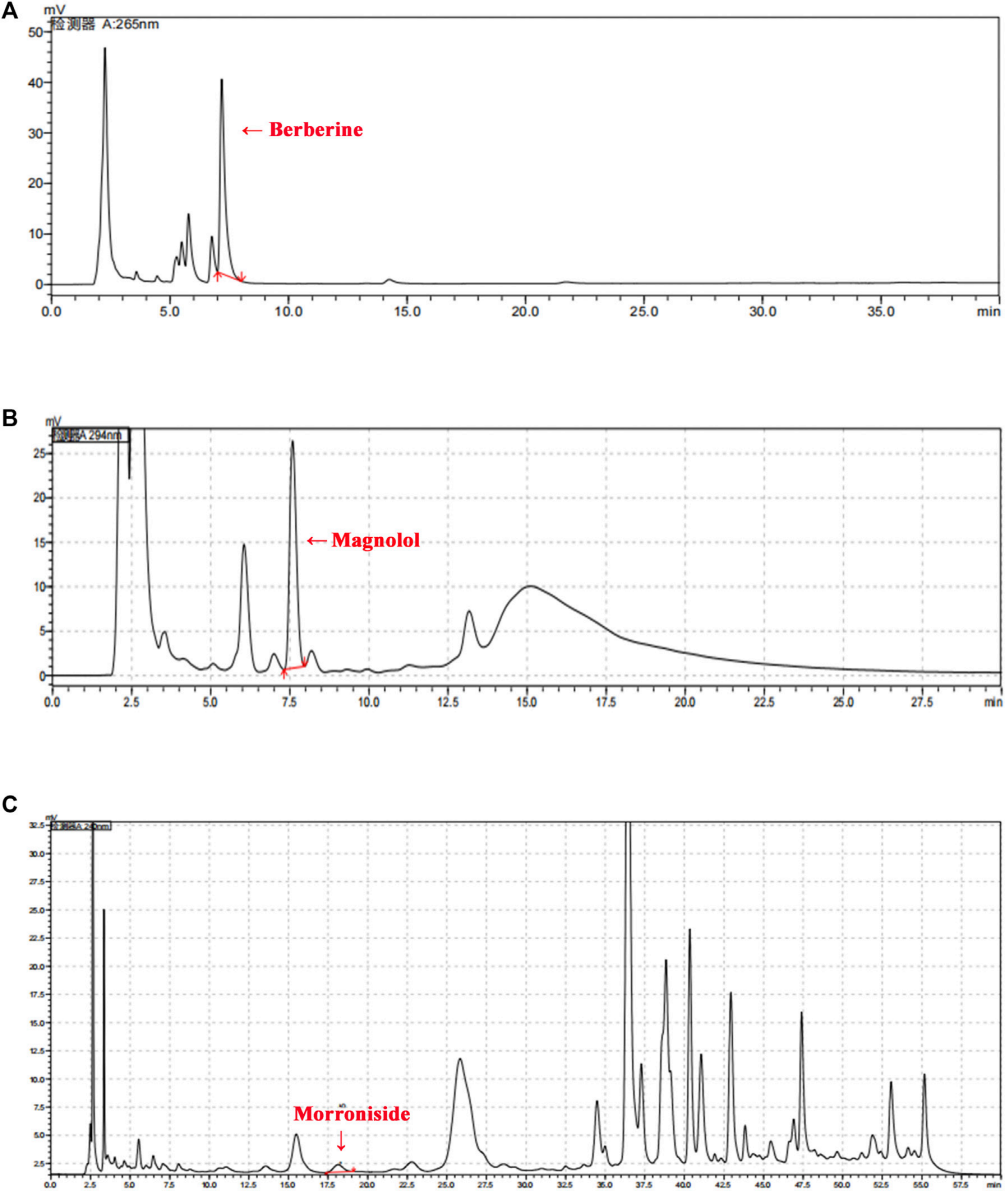
HPLC chromatogram of SHTXF. Three major compounds (berberine, magnolol, and morroniside) of SHTXF were identified and compared with the standards using HPLC. The peaks of berberine [**(A)**, 26 nm], magnolol [**(B)**, 294 nm], and morroniside [**(C)**, 240 nm] are indicated by red arrows.

The Sanghuang Tongxie Formula (SHTXF) is composed of 10 herbal medicines, including *Morus alba L.* [Moraceae] (Sangbaipi) and *Coptis chinensis Franch.* [Ranunculaceae] (Huanglian), *Magnolia officinalis Rehder and E. H. Wilson* [Magnoliaceae] (Houpo), *Pueraria montana* var. *Thomsoni* (*Benth.*) *M.R.Almeida* [Fabaceae] (Gegen), *Astragalus mongholicus Bunge* [Fabaceae] (Huangqi), *Cornus officinalis Siebold & Zucc*. [Cornaceae] (Shanzhuyu), *and Raphanus raphanistrum subsp. Sativus* (*L.*) *Domin* [Brassicaceae] (Laifuzi), *Anemarrhena asphodeloides Bunge* [Asparagaceae]; Zhimu, *Polygonatum odoratum (Mill.) Druce* [Asparagaceae] (Yuzhu), and *Atractylodes lancea* (*Thunb.*) *DC*. [Asteraceae] (Cangzhu) (Figure 1; Table 1). To examine the quality of SHTXF, three representative compounds were selected as the quality standards. When the SHTXF high-performance liquid chromatography (HPLC) chromatogram was compared with the standard control (Supplementary Figure S3), we observed distinguished test peaks of berberine, magnolol, and morroniside in the chemical fingerprints of the SHTXF aqueous extract (Figure 2). Additionally, the proportions of the three ingredients determined by HPLC were 1.4%, 0.34%, and 0.022%, respectively (Table 2). This suggests that the prepared SHTXF contains the necessary active components and is suitable for further study.

**TABLE 2:** HPLC analysis of SHTXF representative components.

Sample name	Proportion (%)	Area	Height	Concentration	Concentration units
Berberine	1.4	559,028	38,519	0.01377	mg/ml
Magnolol	0.34	377,717	25,599	0.02767	mg/ml
Morroniside	0.022	28,048	663	0.00176	mg/ml

### Impaired Insulin Signal in the Fat Body Exacerbates Insulin Resistance and Diabetic Phenotypes

**FIGURE 3:**
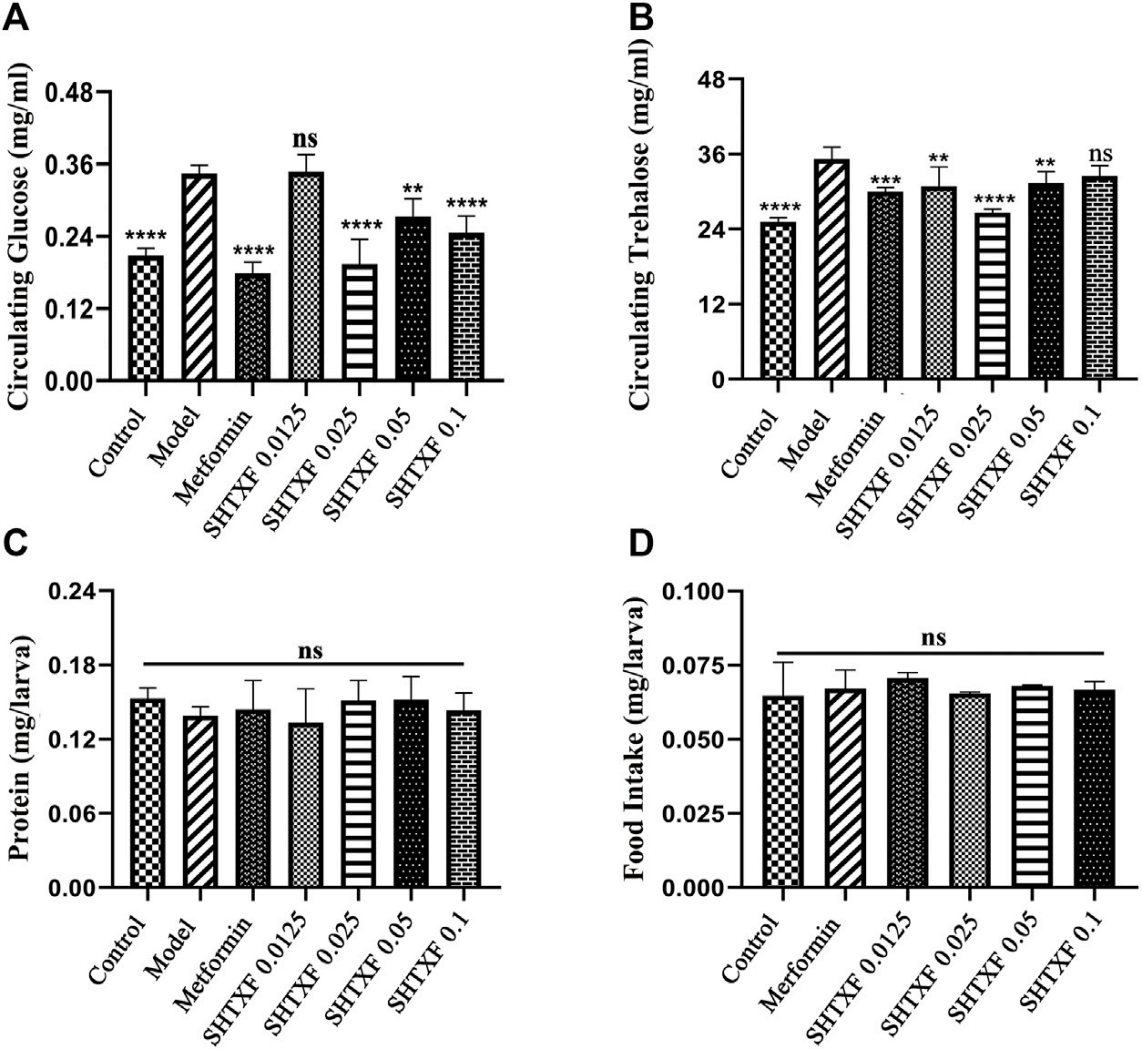
SHTXF reduced the increased level of glucose and trehalose in *Cg* > InR^K1409A^ files. **(A,B)** Assays for circulating the metabolites were performed on the wandering third-instar larvae of indicated groups: hemolymph glucose [**(A)**, 10–12 larvae per pool, *n* = 5], hemolymph trehalose [**(B)**, 6–8 larvae per pool, *n* = 5]. **(C)** Measurement of 20-min food intake by early third-instar larvae as calculated by blue food ingestion (6–8 larvae per pool, *n* ≥ 3). **(D)** Amount of protein indicates the soluble fraction in 0.05% PBST after heat inactivation (3 larvae per pool, *n* = 5). Error bars indicate the standard deviation (SD). One-way ANOVA with Bonferroni’s multiple comparison test was used to compute the *p* values: ****p < 0.0001, ***p < 0.001, and **p < 0.01. ns, no significant difference.

To better dissect the effect of drugs on IR, we employed the GAL4/upstream activation sequence (Gal4/UAS) system and generated an IR model in *Drosophila* third-instar larvae (Brand and Perrimon, 1993), which was achieved by expressing a dominant-negative (DN) form of *Drosophila* InR (carrying the amino acid replacement K1409A, InR^K1409A^, FBtp0018596, http://flybase.org/reports/FBtp0018596) under the control of the fat body (equivalent to mammalian adipose and liver tissues)-specific *Collagen* (*Cg*) promoter (FBti0027802, https://flybase.org/reports/FBti0027802) (Wu et al., 2005; Hennig et al., 2006). Misexpression of the InR antimorphic allele (*UAS*-InR^K1409A^)-driven *Cg*-Gal4 (*Cg* > InR^K1409A^) interfered with the physiological function of endogenous InR and undermined the downstream response of the insulin signaling cascade. As a result, both the hemolymph glucose level and the hemolymph trehalose (a glucose disaccharide that constitutes the major form of circulating sugar in insects) were significantly increased by 67.57% and 40.00%, respectively, in the larval stage (Figures 3A,B, Supplementary Figure S2). This suggests that fat body impairment in insulin signaling triggers insulin resistance (IR) and diabetic phenotypes.

### Sanghuang Tongxie Formula Ameliorates Diabetic Phenotypes of *Cg* > InR^K1409A^ Flies

To determine whether there was an inhibitory effect, we prepared feeding media consisting of SHTXF aqueous extract and raised flies with genotype *Cg* > InR^K1409A^ from the egg stage to the third-instar larval stage. The results showed that the dominant-negative InR-induced elevated circulating glucose was significantly suppressed by SHTXF extract at the concentrations of 0.025, 0.05, and 0.1, but not 0.0125 g/ml (Figure 3A). For circulating trehalose, SHTXF at a concentration of 0.025 g/ml displayed a stronger inhibitory effect than 0.0125 and 0.05 g/ml, while the concentrations of 0.1 g/ml did not show an obvious inhibitory effect (Figure 3B). Next, to exclude the possibility that changes in feeding rate may interfere with the metabolic phenotypes, we evaluated the level of food intake and observed a normal ingestion rate of drug-addled groups or compared with that of the control group (Figure 3C).

The protein content of larvae in each group was similar, with no significant statistical difference (Figure 3D). In addition, the hyperglycemic diabetic phenotypes of *Cg* > InR^K1409A^ larvae were notably inhibited by treatment with metformin (10 mM, a positive control) (Shirazi et al., 2014), which is one of the most popular oral glucose-lowering medications and is widely considered to be the optimal initial therapy for patients with T2DM (Figures 3A,B) (Lv and Guo, 2020). Collectively, SHTXF displayed a dose-dependent effect on *Cg* > InR^K1409A^-triggered diabetic high circulating sugar in the *Drosophila* larval stage.

### Sanghuang Tongxie Formula Rescues the Insulin Resistance-Induced Lipid Homeostasis Disorder

Consistent with the widely investigated roles of the insulin pathway in promoting anabolism, the downregulation of insulin signal activity by expressing InR^K1409A^ in the fat body reduced the total body storage sugar glycogen level by 17.48% during development compared with the *Cg*-Gal4/+ genetically matched controls (Supplementary Figure S4). However, we found that neither SHTXF (0.0125 and 0.025 g/ml) nor metformin significantly altered the overall larval glycogen content in the *Cg* > InR^K1409A^ background. Intriguingly, SHTXF extract at a concentration of 0.05 g/ml or 0.1 g/ml even decreased the level of stored glycogen (Supplementary Figure S4).

**FIGURE 4:**
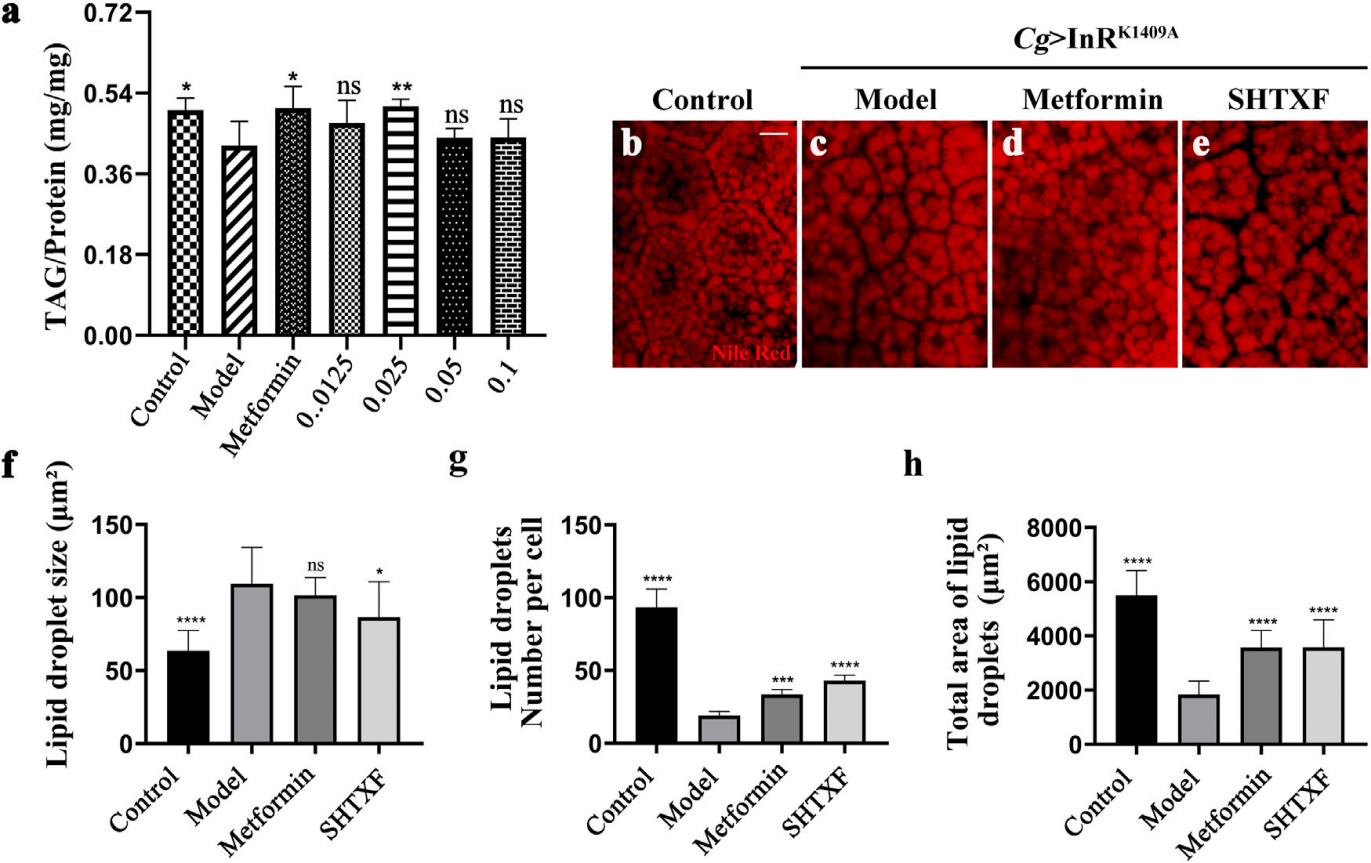
SHTXF rescues the IR-induced lipid homeostasis disorder. **(A)** Assays for stored triglycerides (TAG) contents of *Drosophila* larval stage, which are presented relative to the protein contents of the tissue sample to normalize the weight difference between control and other indicated groups (3 larvae per pool, *n* = 5). **(B–E)** Nile red staining of lipid storage droplets in the larval fat body adipose tissue. **(F–H)** Quantification of lipid droplet size **(F)**, lipid droplets number **(G)**, and total area of lipid droplets in *Cg*-Gal4/+ (control) larvae and *Cg* > InR^K1409A^ larvae in normal, metformin-, or SHTXF-added diets (*n* = 10). The error bars indicate the standard deviation. *p* values were calculated by one-way ANOVA followed by Bonferroni’s multiple comparison test: ****p < 0.0001, ***p < 0.001, and *p < 0.05. ns, no significant difference. Scale bar, 20 µm **(B–E)**.

To examine the effect of SHTXF on lipid metabolism, we first checked the whole body TAG content and found that fat body showed low activity of the insulin pathway (*Cg* > InR^K1409A^) decreased the storage lipid TAG level by 15.67% compared with the control (Figure 4A). Moreover, the reduced TAG level in the IR model was restored by treatment with metformin or 0.025 g/ml SHTXF (Figure 4A). Taken together, these data indicate that SHTXF may contribute toward carbohydrate clearance from the circulatory system by converting it into TAG, not glycogen, for nutrient storage. Ultimately, a concentration of 0.025 g/ml for the SHTXF water extract was chosen, which showed a strong inhibitory effect, to further explore the mechanisms.

To further determine the role of SHTXF in modulating lipid storage, we analyzed the distribution and lipid content in the fat body visualized by Nile Red staining. Consistent with a previous study (Fischer et al., 2017; Lourido et al., 2021), we observed that *Cg* > InR^K1409A^-promoted IR enlarged the fat body cell lipid droplet size and reduced the lipid droplet number, total area of lipid droplets, and cell size when compared with the control (Figures 4B,C,F–H; Supplementary Figure S5). SHTXF partially reversed the increase in the size of lipid droplets and the decrease in lipid droplet number, total area, and cell size (Figures 4E–H; Supplementary Figure S5). Except for lipid droplet size, metformin displayed a similar effect on IR-mediated lipid storage disorder as SHTXF (Figures 4D,F–H; Supplementary Figure S5). These results indicate that SHTXF reverses insulin resistance-induced lipid homeostasis.

### Sanghuang Tongxie Formula Improves the Activity of PI3K

**FIGURE 5:**
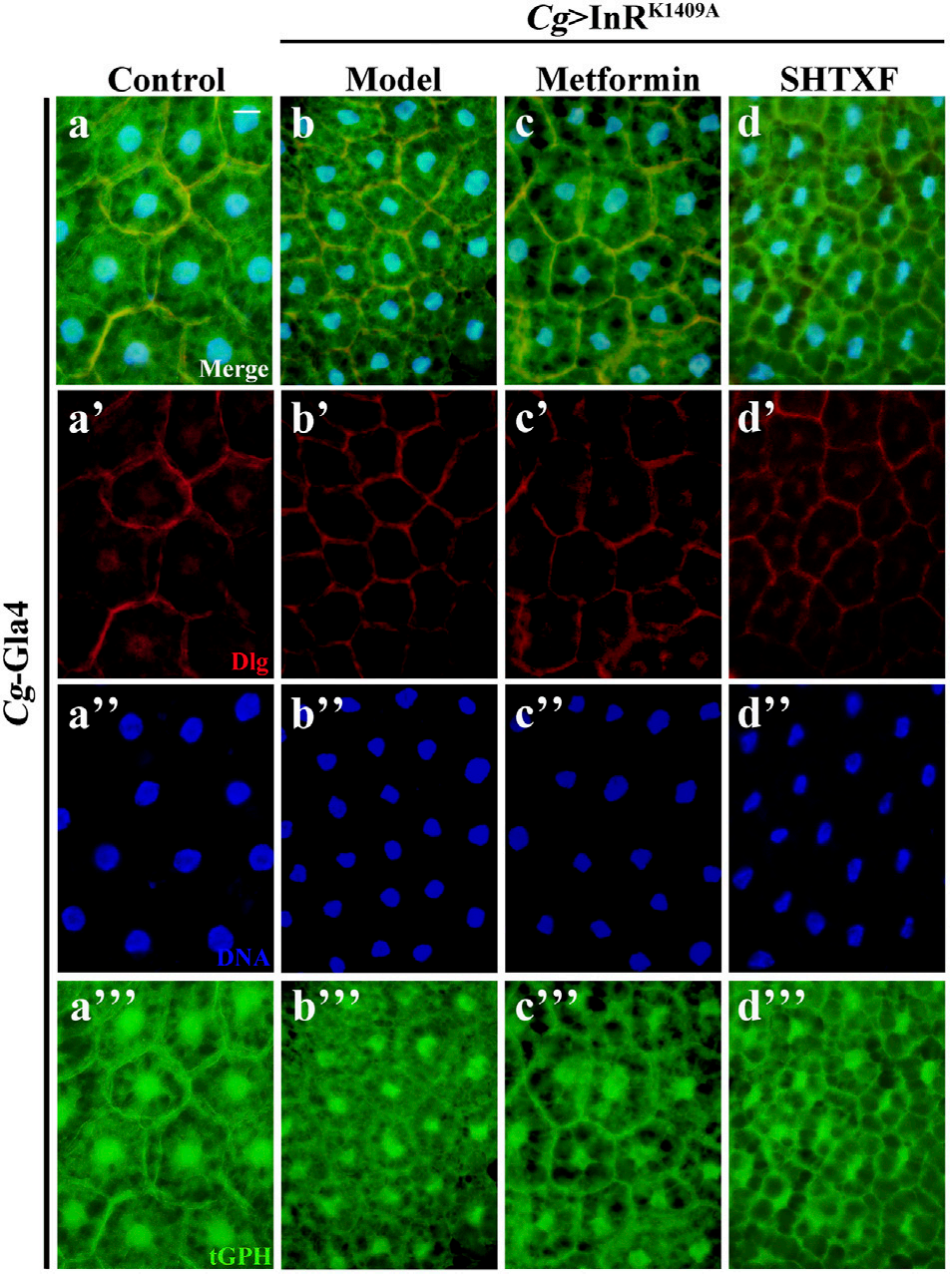
SHTXF improves the PI3K activity *in vivo*. The representative merged fluorescence micrographs show the third-instar larval fat body carrying the tGPH reporter stained with anti-Dlg, which outlines the cell membranes. The individual channels detect only Dlg signal [red, **(A’–D’)**], and only tGPH [green, **(A’’’–D’’’)**]. Nuclei were labeled with DAPI [blue, **(A’–D’)**]. Scale bar, 20 μm. Genotypes: **(A)** *Cg*-Gal4/+; tGPH/+ and **(B–D)** *Cg-*Gal4/+; tGPH/*UAS*-InR^K1409A^.

As phosphoinositide 3-kinase (PI3K) plays a pivotal role in the insulin pathway, we introduced an *in situ* fluorescent reporter termed tGPH (a fusion protein containing *a tubulin* promoter, GFP, and PH domain) (Britton et al., 2002), which recognizes PI (3,4,5)P_3_ and visualizes PI3K activation by observing cell membrane-associated GFP, to check whether insulin signaling was truly impaired in IR diabetic files and the effect of SHTXF. Consistent with our previous work, compared with the control, the fat body cells of the IR model showed decreased cell membrane localization, indicating reduced PI3K activity and the downstream signaling cascade (Figures 5A–A’’’,B–B’’’). While *Cg* > InR^K1409A^ flies were raised on metformin- or SHTXF-added medium, the GFP signal of fat body cells was recruited to the membrane, indicating that PI3K activity was restored (Figures 5C–C’’’,D–D’’’). These results indicate that SHTXF may improve PI3K activity to ameliorate insulin resistance.

### Sanghuang Tongxie Formula Enhances the Phosphorylation Level of Akt

**FIGURE 6:**
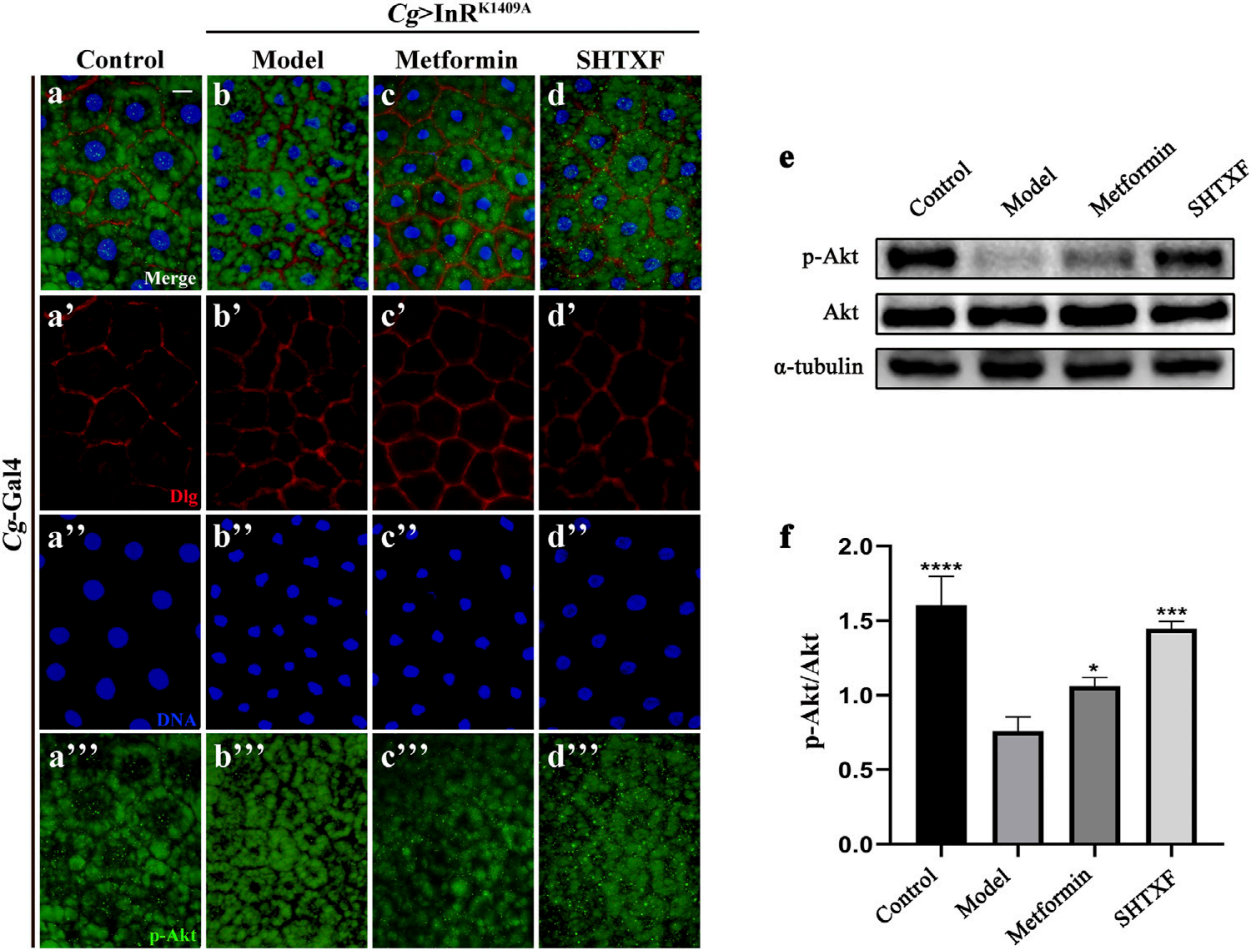
SHTXF increases the level of Akt phosphorylation. **(A–D)** Merged fluorescence micrographs of *Drosophila* third-instar larval fat body are shown. The individual channels detect only Dlg [red, **(A’–D’)**], only DAPI [blue, **(A’’–D’’)**], and only p-Akt [green, **(A”’–D”’)**, fluorescent dots]. **(E)** Western blot analysis of whole-body extracts from third-instar larvae for phosphorylated Akt, total Akt, and α-tubulin (9 larvae per time). α-tubulin was used as a loading control. Groups (from left to right): Control (*Cg*-Gal4/+), Model (*Cg*-Gal4/+; *UAS*-InR^K1409A^/+), Metformin (*Cg*-Gal4/+; *UAS*-InR^K1409A^/+ files treated with 10 mM metformin), SHTXF (*Cg*-Gal4/+; *UAS*-InR^K1409A^/+ files treated with 0.025 g/ml SHTXF). **(F)** Statistical analysis of the p-Akt/Akt relative level from three independent experiments is shown in **(E)** (*n* = 3). The error bars indicate the standard deviation. One-way ANOVA with Bonferroni’s multiple comparison test was used to compute *p* values: ****p < 0.0001, ***p < 0.001, and *p < 0.05. Scale bar, 20 μm **(A–D)**. Genotypes: **(A)** *Cg*-Gal4/+ and **(B–D)** *Cg-*Gal4/+; *UAS*-InR^K1409A^/+.

To further identify the effect of SHTXF on insulin resistance, we monitored Akt phosphorylation using an *in vivo* immunostaining assay and a western blotting (WB) assay *in vitro*. As expected, the misexpression of InR^K1409A^ in the larval fat body (*Cg* > InR^K1409A^) led to reduced Akt Ser505 (equivalent to Ser473 in human Akt1) phosphorylation (p-Akt) in this organ compared with the control (Figures 6A–A’’’,B–B’’’,E,F), while total Akt levels were unchanged (Figure 6E; Supplementary Figure 6A–A’’’,B–B’’’). The decreased p-Akt level in *the Cg* > InR^K1409A^ IR model was largely rescued by SHTXF or moderately inhibited by metformin (Figures 6C–C’’’,D–D’’’,E,F). However, the expression of total Akt protein was unaffected by SHTXF or metformin (Supplementary Figures S6C–C’’’,D–D’’’). Taken together, these data confirmed that SHTXF enhanced Akt phosphorylation.

### Sanghuang Tongxie Formula Blocks dFoxO Transcriptional Activity

**FIGURE 7:**
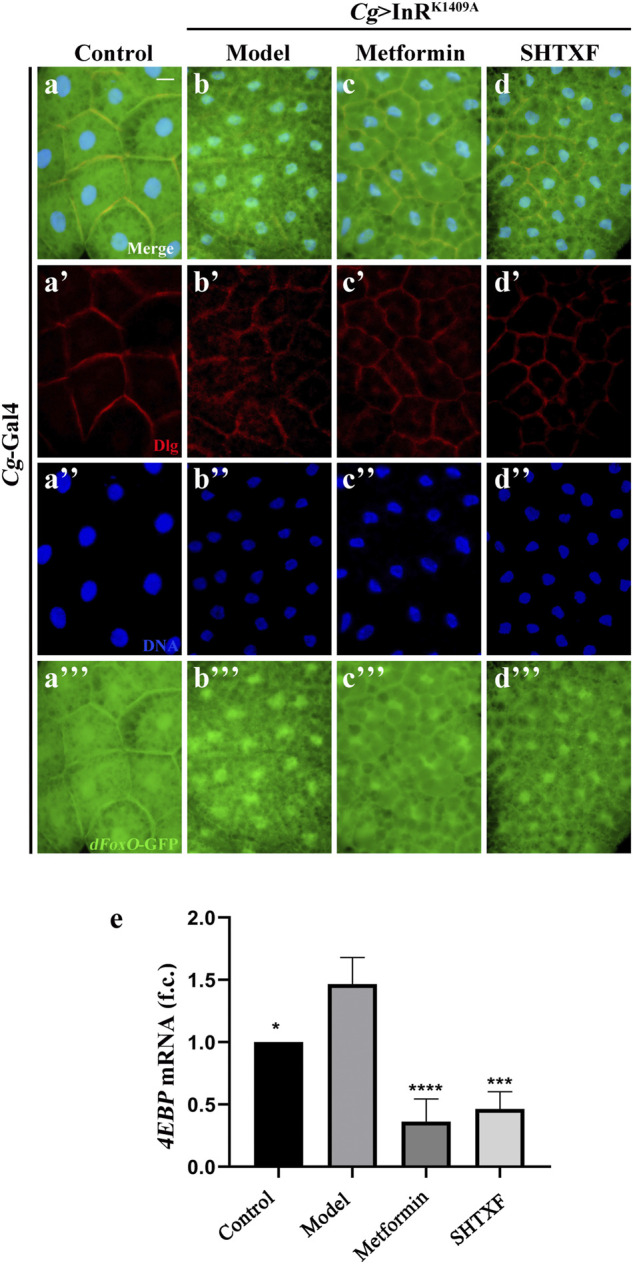
SHTXF remedies *Cg* > InR^K1409A^-triggered dFoxO nuclear localization and transcriptional activity. **(A–D)** Representative merged fluorescence micrographs showing the third-instar larval fat body carrying *dFoxO*-GFP reporter. The individual channels detect only Dlg [red, **(A’–D’)]**, only DAPI [blue, **(A’’–D’’)**], and only *dFoxO*-GFP [green, **(A”’–D”’)**]. **(E)** Histogram shows the *4EBP* mRNA transcription level as measured by qRT-PCR. Total RNA of *Drosophila* third-instar larvae was extracted and normalized for cDNA synthesis. Groups (from left to right): control (*Cg*-Gal4/+), model (*Cg*-Gal4/+; *UAS*-InR^K1409A^/+), metformin (*Cg*-Gal4/+; *UAS*-InR^K1409A^/+ files treated with 10 mM metformin), SHTXF (*Cg*-Gal4/+; *UAS*-InR^K1409A^/+ files treated with 0.025 g/ml SHTXF) (*n* = 3). The error bars indicate the standard deviation from three independent experiments. The *p* values were calculated by one-way ANOVA and Bonferroni’s multiple comparison test: ****p < 0.0001, ***p < 0.001 and *p < 0.05. Scale bar, 20 μm **(A–D)**. Genotypes: **(A)** *Cg*-Gal4/+; *dFoxO*-GFP/+ and **(B–D)** *Cg-*Gal4/+; *dFoxO*-GFP/*UAS*-InR^K1409A^.

To address whether SHTXF modulates the nuclear–cytoplasmic shuttling of *Drosophila* FoxO (dFoxO), we examined the subcellular localization of the *dFoxO*-GFP fusion protein in fat body cells (Wu et al., 2015). We observed that the nuclear localization of *dFoxO*-GFP was significantly increased when InR^K1409A^ was expressed in fat body cells by *Cg*-Gal4 (Figures 7A–A’’’,B–B’’’). SHTXF, similar to metformin, hindered the nuclear localization of *dFoxO*-GFP (Figures 7C–C’’’,D–D’’’). In addition, to monitor dFoxO activity directly, we detected the transcription of the well-characterized dFoxO target gene *4E-BP* using a quantitative reverse transcription-polymerase chain reaction (qRT-PCR) assay. Compared to the control, the upregulated *4E-BP* mRNA level in *Cg* > InR^K1409A^ larvae was appreciably blocked by SHTXF and metformin (Figure 7E), indicating that SHTXF promotes dFoxO cytoplasmic localization and inhibits its transcriptional activity.

## Discussion

*Drosophila melanogaster* has become an ideal model organism for the study of disorders of glucose and lipid metabolism caused by insulin resistance (IR), thanks to the presence of evolutionarily conserved metabolic signaling pathways (insulin and insulin-like growth factor signaling pathways) and hormone regulation processes (the antagonistic regulation of insulin-like peptides and adipokine hormones). More importantly, the successful application of the GAL4/UAS binary expression system has made it possible to reveal complex gene functions (Musselman et al., 2018). As only one InR and insulin receptor substrate (IRS, encoded by chico) is expressed in *Drosophila*, we employed the GAL4/UAS system to specifically express the dominant-negative form of InR (*UAS*-InR^K1409A^) in the fly fat body (*Cg*-Gal4) by genetic hybridization (Supplementary Figure S2). Logically, the misexpression of the InR antimorphic allele interferes with the physiological functions of endogenous InR and reduces the transduction activity of insulin signaling, resulting in IR, and establishing the *Cg* > InR^K1409A^ diabetic model. A previous study reported that a high-sugar diet (HSD) could induce obesity and insulin resistance in wild-type *Drosophila* with type 2 diabetes (Pasco and Léopold, 2012). However, the *Cg* > InR^K1409A^ IR model generated in our study did not present an adiposity phenotype with reduced total TAG content (Figure 4A). Given the complicated relationship between fat and IR during diabetes occurrence and development, applying the *Cg* > InR^K1409A^ model could better elucidate the roles of IR in diabetes and screen potential drugs or ingredients.

The sugars in *Drosophila* mainly participate in nutrient circulation in the form of glucose (monosaccharide) and trehalose (disaccharide), provide energy for growth and development through glycolysis, and store energy in the form of glycogen (Heydemann, 2016). As a major nutrient, lipids store energy, participate in cell membrane structure, and synthesize hormones and vitamins, which are essential for normal life activities. When apolipoprotein binds to its receptor, triglycerides (TAG) are generated and enter the body’s adipocytes to form lipid droplets and organelles which are common in animal and plant cells that store neutral fat (Heier and Kühnlein, 2018). Therefore, we analyzed glucose and lipid metabolism in fruit flies fed SHTXF aqueous extract. As a result, it was found that SHTXF may contribute to sugar clearance from the circulating system by transforming it into TAG, but not glycogen, for nutrient storage. Moreover, our results showed that SHTXF could improve the activity of PI3K, promote the phosphorylation of Akt, and impede dFoxO transcriptional activity. Overall, these results indicate that SHTXF activates the central factors of the PI3K/Akt signaling pathway to ameliorate IR.

As a self-drafted patent prescription based on TCM theory and clinical experience, SHTXF contains 10 herbs and has shown a notable effect on the treatment of IR diabetes with its active ingredients (Chinese invention patent No. ZL201510724687.8). *Coptis chinensis Franch.* [Ranunculaceae] (Huanglian) has been used for centuries as an anti-diabetic drug in TCM, and its main bioactive base berberine can reduce blood glucose, regulate lipids, and improve insulin resistance to alleviate T2DM (Abd El-Wahab et al., 2013). Berberine has been found to show potential in acting on block lipid transport-1 (BLT1), modulating the leukotriene B4-Block lipid transport-1 (LTB4-BLT1) axis to ameliorate insulin resistance and inflammation. It has also been found to increase the peroxisome proliferator-activated receptor γ co-activator 1α (PGC1α), activate the phosphorylation of adenosine monophosphate (AMP), protein kinase (p-AMPK), and liver kinase B1 (p-LKB1), and reduce the adenosine monophosphate/adenosine triphosphate (AMP/ATP) ratio rate, thereby reversing fructose-induced insulin resistance (Li et al., 2020; Gong et al., 2021). In addition, magnolol exerts antioxidant activity by inhibiting the liver activity of cytochromeP450, family2, subfamily E, and polypeptide1 (CYP2E1) to improve glucose tolerance, thereby reducing IR and improving glucose and lipid metabolism disorders (Wang et al., 2014). *Cornus officinalis Siebold & Zucc.* [Cornaceae] extract can promote the expression of peroxisome proliferator-activated receptor γ (PPAR-γ) in adipocytes, increase the expression of recombinant glucose transporter 4 (GLUT4) and adiponectin, improve insulin resistance, and increase glucose utilization, ultimately achieving the effect of lowering the blood sugar levels (Kim et al., 2009). Thus, SHTXF treatment in diabetic IR appears to be the result of multiple factors, multiple pathways, and multi-target regulatory networks.

Based on previous research, our work focuses on the positive effect of SHTXF on IR by modulating the InR/PI3K/Akt signaling pathway, not only indicating potential drug targets but also providing key insights for the clinical treatment of T2DM and related metabolic diseases.

## Data Availability

The original contributions presented in the study are included in the article/Supplementary Material, further inquiries can be directed to the corresponding authors.
