# Supplementary material for: Sanghuang Tongxie Formula Ameliorates Insulin Resistance in Drosophila Through Regulating PI3K/Akt Signaling
Source: Front Pharmacol. 2022 Jun 6;13:874180. doi: 10.3389/fphar.2022.874180 (PMC9207506; doi:10.3389/fphar.2022.874180)
Supplement: Supplementary file 1 [file Presentation1.pdf]

Sanghuang Tongxie Formula ameliorates Insulin Resistance in *Drosophila* through regulating PI3K/Akt Signaling

Xuqing Cao[1#], Xiaojin La[1#], Biwei Zhang[1], Zhigang Wang[1], Yinghong Li[1], Yanping Bo[1], Hong Chang[1], Xiujuan Gao[1], Chunyu Tian[1], Chenxi Wu[1*] and Ji-an Li[1*]

[1] Hebei Key Laboratory of Integrated Traditional Chinese and Western Medicine for Diabetes and Its Complications, College of Traditional Chinese Medicine, North China University of Science and Technology, 21 Bohai Road, Tangshan 063210, China

[#] These authors contribute equally to this work.

[*]Correspondence should be addressed to:chenxi.wu@ncst.edu.cn, lnyy@vip.sina.com

Supplementary materials

Cao *et al*., Figuer S1

a

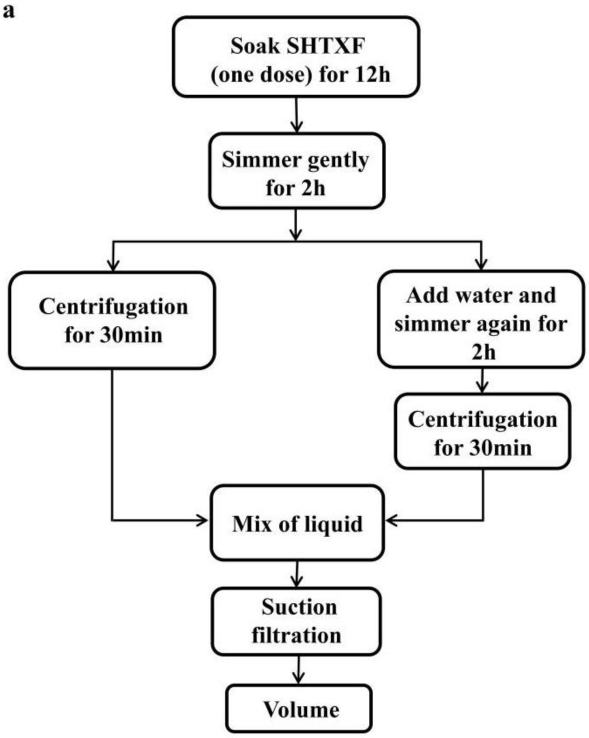

Figure S1. Preparation process of SHTXF Extract.

Outline of the SHTXF extract preparation.

Cao *et al.*, Figuer S2

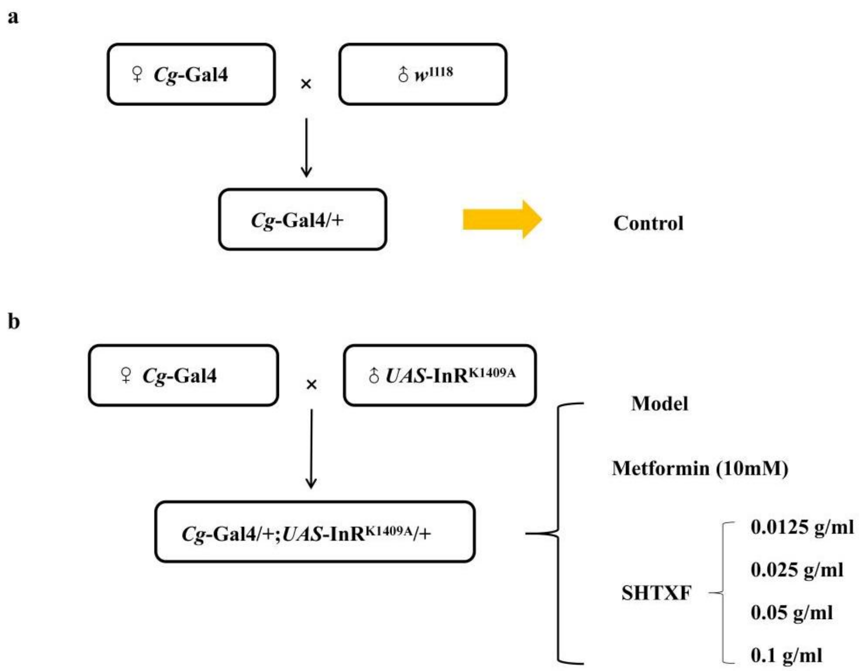

Figure S2. Crossing scheme for experiment groups.

Healthy unmated female flies with genotype *Cg*-Gal4 (Chromosome 2) were crossed to male flies with genotype w^{1118}, and collect their offspring which were reared in normal medium (supplemented with sucrose, agar, corn meal and yeast) for control group (**a**). Healthy unmated female flies with genotype *Cg*-Gal4 were crossed to male flies with genotype *UAS*-InR^{K1409A} (Chromosome 3), and the offspring with genotype *Cg*-Gal4/+; *UAS*-InR^{K1409A}/+ were kept in regular medium for insulin resistance model group, or reared in the medium added with metformin (10mM) or SHTXF (0.0125g/mL, 0.025g/mL, 0.05g/ml or 0.1g/mL) respectively for drug treated groups (**b**).

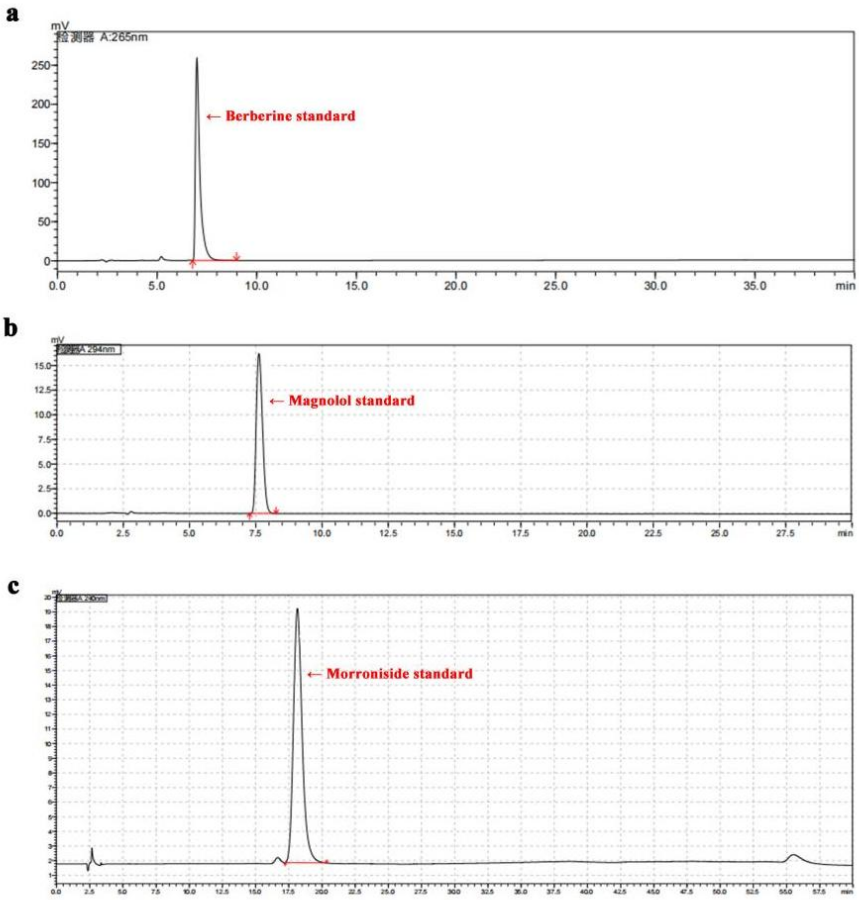

Figure S3. The HPLC chromatogram of standard controls.

The standard of berberine, magnolol and morroniside were identified by HPLC method. The detection wavelengths of berberine (**a**), magnolol (**b**) or morroniside (**c**) is 265nm, 294nm or 240nm respectively, where their peaks indicated with colored arrows.

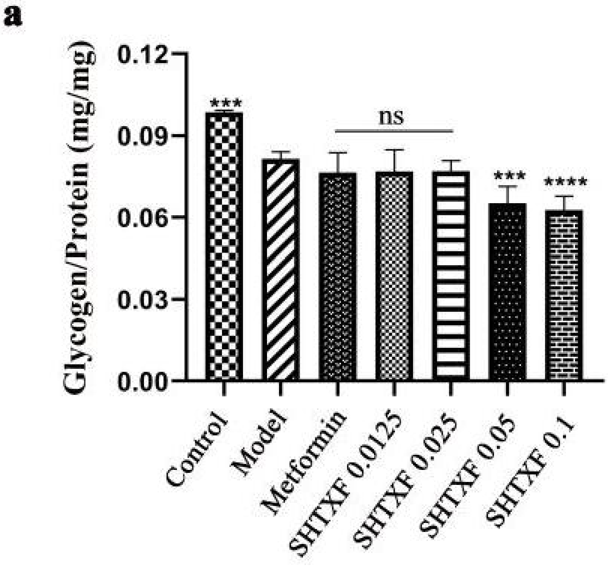

Figure S4. SHTXF could not rescue glycogen storage.

Glycogen content of the indicated *Drosophila* groups at the third-instar larval stage (3 larvae per pool, n=5). *P* values were calculated by one-way ANOVA and Bonferroni's multiple comparison test: **** $P<0.0001$, *** $P<0.001$; ns: no significant difference.

a

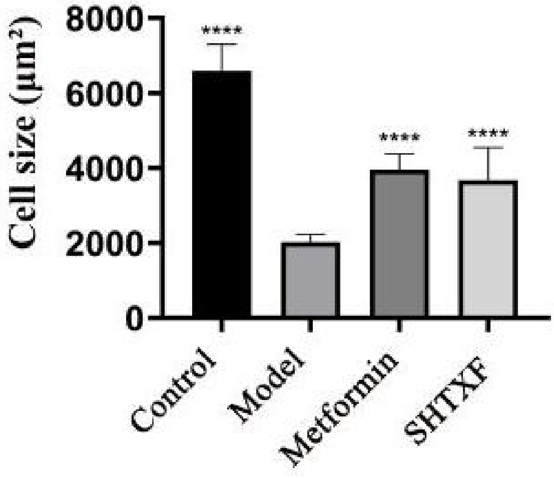

Figure S5. SHTXF reserves the reduced cell size.

Cell size of the indicated *Drosophila* groups at the third-instar larval stage (n=10). *P* values were calculated by one-way ANOVA and Bonferroni's multiple comparison test: **** $P<0.0001$.

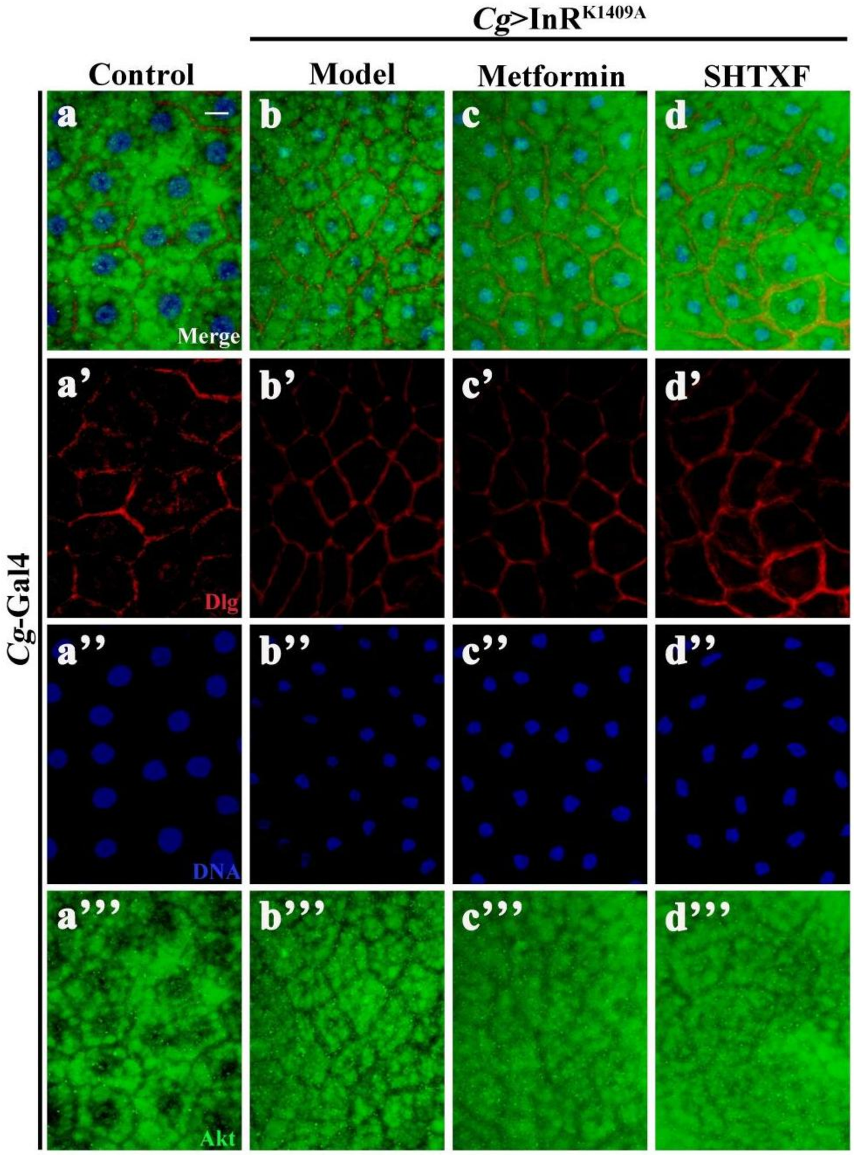

Figure S6. SHTXF does not change the expression of total Akt protein.

(a-d) Merged fluorescence micrographs of *Drosophila* third-instar larval fat body are shown. The individual channels detecting only Dlg (red, a'-d'), only DAPI (blue, a''-d'') and only Akt (green, a'''-c''', fluorescent dots). Scale bar: 20μm.
